# Economic policy uncertainty and presidential approval: Evidence from Latin America

**DOI:** 10.1371/journal.pone.0248432

**Published:** 2021-03-15

**Authors:** Myriam Gómez-Méndez, Erwin Hansen

**Affiliations:** 1 Institute of Political Science, P. Catholic University of Chile, Santiago, Chile; 2 Department of Business Administration, Faculty of Economics and Business, University of Chile, Santiago, Chile; Georgia State University, UNITED STATES

## Abstract

This paper analyzes the extent to which economic policy uncertainty affects presidential approval in four Latin American countries (Brazil, Chile, Colombia, and Mexico). Using panel (time-series cross-sectional) estimation methods, we show that economic policy uncertainty has a negative impact on presidential approval in our sample. A one-standard-deviation increase in the level of economic uncertainty reduces presidential approval by approximately 12 percent. Our results are consistent with the political economy model of Alesina et al. (1993), which shows that voters are less likely to re-elect the incumbent when faced with uncertainty about economic policy. Incumbent competence signalling can exarcerbate this effect.

## 1. Introduction

Theoretical political economy models like those of [1] and [2] have established that there is a strong relationship between government policies and the state of the economy. Extensive empirical testing to determine whether economic variables affect government populatiry has yielded mixed results (see, e.g., [3–5]). Less is known, however, about how economic policy uncertainty affects presidential approval. Economic policy uncertainty may change the behavior of individuals by reducing consumption and that of firms by reducing investment and hiring [6]. Therefore, it is plausible that economic policy uncertainty also affects voters’ perceptions of government performance, and as a consequence, presidential approval ratings.

Literature regarding economic perceptions and presidential approval tends to gravitate towards the study of high-income economies, such as the US [7–9]. These studies provide insight into the determinants of citizens’ approval of the executive branch. According to these analyses, the state of the national economy is frequently one of citizens’ main concerns. Subsequent scholarship demonstrates that the way in which the executive branch manages the economy is an important variable that impacts government approval (e.g., [10–12]). Thus, we argue that economic policy uncertainty influences citizens’ approval of their head of government. Understanding the variables that affect approval ratings is crucial for democratic governments, given that presidential approval is a desirable good for the chief-of-state and their political party [13, 14]. We argue that it is important to understand the relationship between economic policy uncertainty and presidential approval given that the latter is a means to other political ends such as continued party incumbency and the ability to introduce legislation. In the specific case of the US, there is further evidence that high approval ratings have lent the executive the political capital necessary to pass policy, and/or in times of foreign crises, that they have provided enough legitimacy to take the country to war [15].

However, less is known about low-income economies. Only a few studies offer insight into presidential approval dynamics in Latin America (see, e.g., [16–18]). Still, we can attest to the fact that in this region too, governments are often rewarded or punished for their management of the economy. As such, approval ratings supply political capital in these countries as well. For instance, a growing body of literature suggests that economic crises have a direct impact on voters’ behavior [14, 19–21]. Keep in mind that Latin Americans have lived through a series of economic crises since the 1980s in conditions different to those in the US, such as fragmented or weak formal institutions, a lack of adequate checks and balances, hyperinflation, inequality, and poverty, thus to analyze the effects of economic policy uncertainty on presidential approval is relevant.

In Latin America, on the one hand, high approval ratings coupled with strong economic performance have afforded executives disproportionate authority with which to pass policies (by using unilateral decree powers for example [22], to increase their formal powers or even to alter term limits [15, 23, 24]. On the other hand, low approval ratings have been linked to electoral defeats for political parties and even impeachments or early departures, as in Peru and Brazil [20, 21, 23, 25]. Elaborating on these arguments, Singer states that "*[i]t is common to ascribe the rise of the left to the failures of the economic reforms of previous decades and voter frustration with them*" (2013:182). This is a clear suggestion that the state of the economy may have an impact on approval ratings. Building on these analyses, several studies have found that presidential approval in Latin America is significantly associated with fluctuations in both inflation and growth rates [17, 19, 26].

We examine the extent to which economic policy uncertainty affects presidential approval empirically using the economic policy uncertainty (EPU) index recently made available by [27]. The EPU index is a newspaper-based uncertainty index that has been used extensively in recent work to study the impact of uncertainty on macroeconomic aggregates and stock markets. We focus our analysis on four Latin American economies (Brazil, Chile, Colombia, and Mexico) for which presidential approval data is available from the Executive Approval Database (EAD) 1.0 (see [28]). We use panel (time-series cross-sectional) estimation methods to address our main research question. We perform two empirical analyses to evaluate the potential effect of economic policy uncertainty on presidential approval. In the first analysis, we extend [29]’s work examining the effect of policy regimes on economic accountability in Latin America by adding our proxy for economic uncertainty (the EPU index) to the regression modelling presidential approval. In the second analysis, we take a more agnostic view of the causal relationship between the variables of interest and we estimate impulse response functions (IRF) using a Panel-VAR model. The estimated IRF quantify the impact of an economic policy uncertainty shock on presidential approval ratings within a framework in which all the variables in the model are endogenous. The PVAR method is also better suited for capturing the joint dynamics of economic uncertainty and presidential approval and for identifying interlinkages more naturally.

Both empirical analyses show that economic policy uncertainty negatively affects presidential approval. In our first analysis, i.e., the extended version of the model by [29], the estimated coefficient for the EPU index is negative and statistically significant in five out of the six specifications considered. This result is robust to the inclusion of variables controlling for the economic outlook (inflation and growth), honeymoon and scandal effects, the nature of presidential regimes (neoliberalism or statism), and country fixed effects. In our second analysis, the estimated IRF obtained from the PVAR model of the four Latin American economies in the sample show that a one-standard-deviation shock on the EPU index reduces presidential approval by approximately 12 percent. The estimated effect is statistically significant and lasts for about nine months. The PVAR model also include as control variables the industrial production growth rate, the inflation rate, the unemployment rate and the stock market index return, which may be considered as an alternative proxy for uncertainty.

Our results are consistent with the predictions from the theoretical political model of [2]. These authors’ model explicitly accounts for economic policy by allowing the policymaker to set the inflation rate. We argue that an EPU shock can be captured in the model by an inflation surprise brought about by the policymaker. The model predicts that upon observing this shock, the voters will update their beliefs about the incumbent’s competence and penalize them with low re-election chances. We expect this downgrade to translate into low presidential approval ratings. Our empirical results show that following an EPU index shock, presidential approval decreases significantly, while inflation increases. Furthermore, the model anticipates a second, “indirect” channel through which EPU may affect presidential approval: a negative effect on output growth. Prior literature (see [27]) has found that shocks to the EPU index negatively impact growth. In our sample, and consistent with this idea, we observed that industrial production decreases after an EPU index shock, but that the effect is not statistically significant.

From a theorerical point of view, there is a third possible channel in the [2] to explain our results: the impact of the EPU shock on the competence signal. However, it is relatively unclear how this signal could be affected by the EPU index. [30] argue that voters extract the competence signal by comparing the relative economic performance of the domestic economy with that of the global economy. However, domestic and foreign sources of uncertainty are indistinguishable because of how the EPU index is constructed. [29, 31] discuss a second mechanism to discern between competence and non-political shocks. The authors argue that the competence signal is related to countries’ political regime. They hypothesise that voters believe the policymaker has greater control over the economy in more statist regimes than in more neoliberal ones. A priori, we identify no clear mechanism through which an EPU shock may affect any given country’s political regime, at least in the short run.

Our results contribute to the literature in several respects. First, we provide new empirical evidence regarding the impact of economic policy uncertainty on presidential approval. To the best of our knowledge, this is the first study to use a newspaper-based measure of uncertainty among the literature on economic voting and the determinants of presidential popularity. The few existing studies focussing on this relationship either use stock market volatility [32, 33] or GDP growth volatility [34] as proxies for uncertainty. It is noteworthy that our main result is not explained by proxies for the economic outllok, indicating that there is a difference between the economic uncertainty channel and the more traditional economic outlook channel. This result is consistent with the model by [2], where both channels can be differentiated.

Secondly, our results contribute to the literature on macro-political dynamics. This body of work aims to identify dynamic interlinkages between economic and political variables using time-series estimation methods (see, e.g., [35]; and, [36]). We contribute by using a Panel VAR approach that simultaneously captures the joint dynamics of the variables of interest and uses data from a panel of countries simultaneously. In the literature on macro-political dyanamics single-country studies (usually about the US) are the most common. Thirdly, we contribute to research on the determinants of presidential approval and the estimation of macro-political linkages in Latin America. We add to the literature by incorporating a proxy for economic policy uncertainty and by using a novel empirical method to estimate its potential impact on presidential approval.

The remainder of the paper is structured as follows. In section 2, we provide a theoretical framework for the empirical exercise. In section 3, we describe the data and variables, for which we provide descriptive statistics. In Section 4, we explain our empirical analysis and report our main results. In section 5, we conclude.

## 2. A theoretical framework

We base our empirical exercise on the political economy model of [2]. The model jointly determines economic growth and national election results using a framework with rational, forward-looking voters and governments, with both parties interacting sequentially. The model explicitly incorporates economic policy by allowing the government to define the inflation rate. Given this feature, it is relatively straightforward to identify a channel through which EPU can affect the voters’ approval of the incumbent. This relationship is precisely the focus of our empirical study, in which we measure the two terms using the EPU index and the presidential approval rate, respectively.

We introduce the basic elements of the model. The supply side of the economy is characterized by the following equation:

$$g_{t}=\bar{g}+\gamma (\pi_{t}-\pi_{t}^{e})+\varepsilon_{t},$$ (1)

where *g*_*t*_ is the output growth, $\bar{g}$ is the natural rate of growth, *π*_*t*_ is the inflation rate, and $\pi_{t}^{e}=E(\pi_{t}|I_{t-1})$ is the expected inflation rate given the information set, *I*_*t*−1_. The error term *ε*_*t*_ has two components between which the voters cannot distinguish: a transitory shock capturing unanticipated economic shocks unrelated to government actions, *ξ*_*t*_, and a shock capturing government competence, *η*_*t*_:

$$\varepsilon_{t}=\xi_{t}+\eta_{t}.$$ (2)

The transitory shock, *ξ*_*t*_, is i.i.d with mean 0 and variance $\sigma_{\xi }^{2}$. [2] interpreted the competence shock as the administration’s ability to avoid inefficiency and to create an environment conducive to growth without inflation. The model assumes that there is inertia in the competence shock by modelling it as a first-order moving average process, as follows:

$$\eta_{t}=\mu_{t}+\rho \mu_{t-1}.$$ (3)

The competence shock has mean 0 and variance $\sigma_{\mu }^{2}.$

Voters’ preferences are captured by a utility function, which is quadratic in inflation surprises and linear in output growth:

$$W^{i}=\overset{\infty }{\underset{t=0}{\sum }}\beta^{t}\left[-\frac{1}{2}{\left(\pi_{t}-{\bar{\pi }}_{t}^{i}\right)}^{2}+b^{i}g_{t}\right],$$ (4)

where the index *i* represents a particular voter (or political party in the original model of [2]). All voters value output growth but they prefer different rates of inflation and hold differing expectations regarding the trade-off between output and deviations of inflation from its preferred level (*b*). The only economic policy the model accounts for is the inflation rate set by the policymaker. The model assumes that policymakers have direct control over the inflation rate, in a way that is consistent with voters’ rational expectations. [30] use the same configuration, for example, and assume that the policymaker adjusts the inflation rate to voters’ expectations. In this more general specification, the policymaker may decide to generate unexpected inflation. This key aspect of the model allow us to captur the effect of EPU on an incumbent’s re-election chances, proxied by presidential approval rates in our empirical exercise.

Since the voters are understood to benefit from growth, the model assumes that they will prefer incumbents with above-average competence to remain in office. Assuming that the voters observe *g*_*t*_, $\bar{g}$, *π*_*t*_, $\pi_{t}^{e}$, and *μ*_*t*−1_, [2] show that the optimal forecast of the incumbent’s post-electoral competence is given by:

$${\hat{\eta }}_{t+1}=\left[g_{t}-\bar{g}-\gamma (\pi_{t}-\pi_{t}^{e})-\rho \mu_{t-1}\right]\rho \left(\frac{\sigma_{\mu }^{2}}{\sigma_{\mu }^{2}+\sigma_{\xi }^{2}}\right).$$ (5)

The voters’ decision depends on the output gap, $\left(g_{t}-\bar{g}\right)$, the inflation surprise, ${(\pi }_{t}-\pi_{t}^{e})$, and the incumbent’s past record of competence, (*μ*_*t*−1_), while adjusting for the persistence of the competence shock, (*ρ*), as well as the ratio between the government’s competence shock and the transitory shock unrelated to the government actions: $\left(\frac{\sigma_{\mu }^{2}}{\sigma_{\mu }^{2}+\sigma_{\xi }^{2}}\right)$. This ratio quantifies the voters’ ability to extract information about the incumbent’s competence from the state of the economy. If the variance of the transitory shock reaches 0, the ratio converges to 1. In such a case, the economy’s growth and rate of inflation would be understood to have been affected by the incumbent’s competence, rather than by non-political shocks. The higher the value of $\sigma_{\xi }^{2}$, the more the state of the economy is ascribed to non-political shocks and the lower the clarity of the signal the voters extract regarding the incumbent’s competence.

In this framework, EPU can be conceptualised as the policymaker surprising the voters by setting the inflation rate above the level they expected, to impose a larger tax burden. According to Eq (1), this inflation surprise at time *t*, after being observed by the voters, reduces confidence in the incumbent’s future competence and thus their re-election chances. Of course, the overall effect depends on the competence signal, whose value varies between 0 and 1. If the voters infer that unexpected inflation is due to the policymaker (i.e., when the competence signal is 1), the negative impact on the competence projection will be larger. On the contrary, if the voters infer that part of the unexpected inflation is explained, for example, by a shock in international prices that is battering the local economy, the effect will be lower. Although the way the EPU index is designed does not make it possible to precisely determine whether news items are from external or domestic sources, we believe that it must rely more heavily on the former source and therefore that the competence signal should be significantly different from zero.

Voters’ assessment of the future competence of the incumbent may also be affected by EPU through its direct effect on output growth. Prior literature has found that EPU shocks have transitory, negative effects on output and investment (see, e.g., [27]). Consistent with this empirical evidence, a shock in EPU should be associated with a lower realization of $\left(g_{t}-\bar{g}\right)$ in Eq (1), and therefore lower forecasts of the incumbent’s competence and lower re-election chances.

The political economy model of [2] provides a sound theoretical framework with which to understand the potential effects of EPU shocks on presidential approval. In the model, a shock in EPU occurs when the inflation rate set by the policymaker exceeds voters’ expectations. Upon observing this negative shock, the voters downgrade their forecast of the incumbent’s future competence, thereby reducing electoral approval. An additional channel would be the effect of EPU on output growth. Prior literature has documented a decline in output growth following EPU shocks (see [27]). In this case, the model also predicts that voters will negatively assess the incumbent, thus reducing their electoral approval.

Overall, the model of [2] provides a theoretical framework suggesting that EPU is detrimental to incumbent approval. It clearly identifies at least two channels through which this effect may operate: an unexpected inflation channel and an output growth channel. We test these predictions below.

## 3. Data and variables

We use the following variables in the analysis:

### Economic policy uncertainty

As a proxy for economic uncertainty, we use the Economic Policy Uncertainty (EPU) index of [27]. The EPU index measures uncertainty based on newspaper coverage of key concepts associated with economic adversity and unexpected events. In the US for example, the index incorporates data from ten leading newspapers by counting articles including the keyword triplet "uncertainty" or "uncertain" "economic" or "economy", as well as one of the following terms: "congress", "deficit", "Federal Reserve", "Legislation", or "White House" (see [27]). Thus, the EPU index reflects the number of publications about uncertain, economic, and policy-relevant events.

Monthly EPU data is available at http://www.policyuncertainty.com/ for a large number of countries, including developed and developing countries. Specifically, the EPU index is available for the four Latin American economies considered in this study. Professor Baker and coauthors are responsible for the Brazilian and Mexican indices. The Chilean and Colombian indices are built by [37] and [38], respectively. The four indices are constructed following the methodology used by [27] for the US. EPU data is available for Brazil since 1991, for Chile since 1993, for Colombia since 2000, and for Mexico since 1996.

### Presidential approval

We use presidential approval data from the Executive Approval Database (EAD) 1.0 (see [28]). This dataset provides a comparable metric of presidential approval for 18 Latin American countries, including Brazil, Chile, Colombia, and Mexico, for which the EPU index is also available. The presidential approval series combines presidential popularity ratings from several surveys using alternative methodologies into a single smoothed time series using the procedure in [39]. This database has recently been used in several research projects (see, for example, [23, 29, 31, 40, 41]). The data and further details regarding their construction can be found at http://www.executiveapproval.org/.

### Control variables

Besides economic uncertainty and presidential approval variables, we include additional macroeconomic variables in our empirical analysis to control for the economic outlook. Following the literature (see, e.g., [3]), we include data relating to industrial production, inflation, unemployment, and stock market index returns for each of the economies in the sample. These were sourced from the OECD’s statistical database.

**Fig 1:**
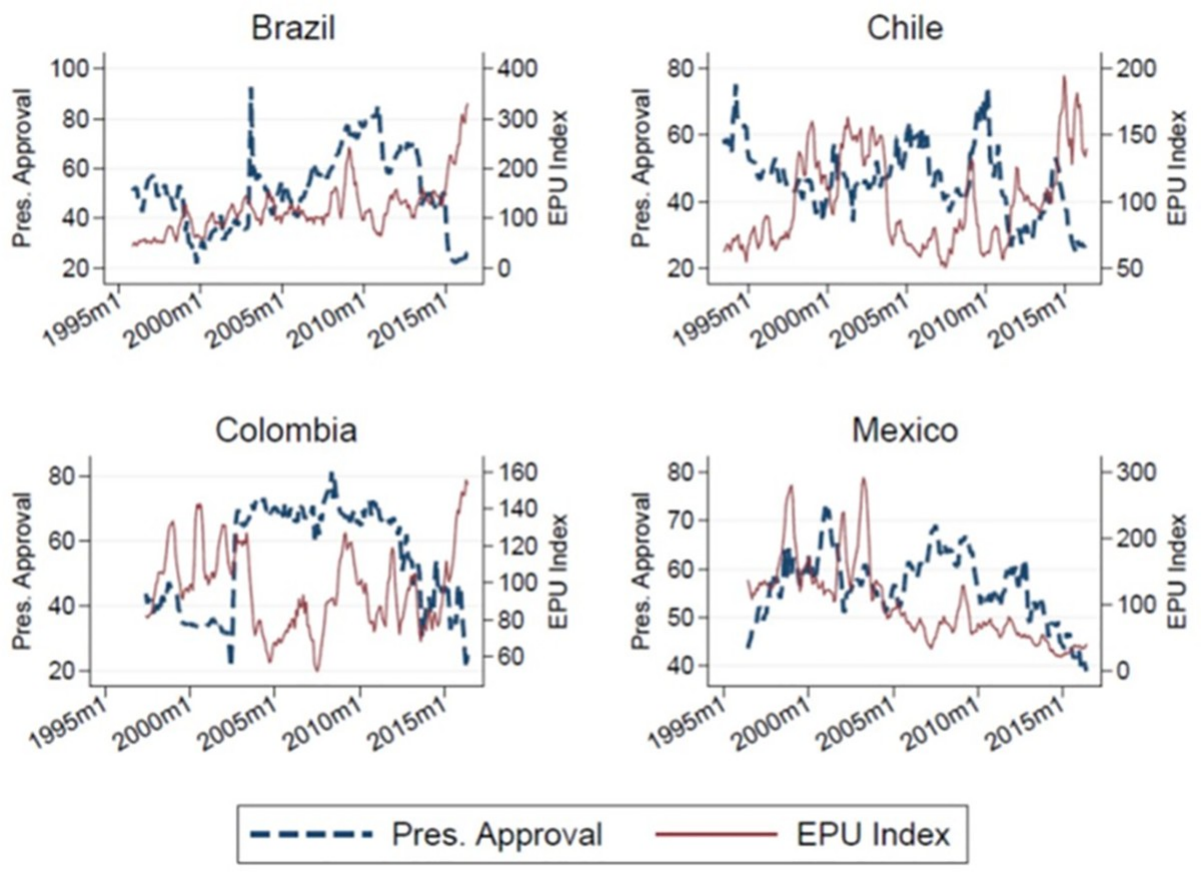
The EPU index and presidential approval ratings over time. This figure provides plots of presidential approval ratings (%) against the monthly time series of the EPU index (6-month moving average), by country.

Fig 1 shows the time series evolution of the EPU index (dashed line) and the presidential approval (regular line) variables for the four countries in the sample. The plots demonstrate some of the series’ noteworthy empirical features. Firstly, both series vary markedly over time and for each country. Unsurprisingly, the uncertainty measure is the more volatile of the two, but the presidential approval series also varies significantly over time. Secondly, and more importantly for this study, we observe that both series tend to move in opposite directions over time. In other words, they seem to be negatively correlated. Nevertheless, some heterogeneity in the observed pattern of these two variables is to be noted. For example, in the case of Brazil, the negative correlation appear stronger in the second part of the sample. In the case of Chile, the pattern is more apparent as we observe an explicit mirroring throughout most of the analyzed period. In Colombia, this pattern is clearer after 2003, while in Mexico, it is also more evident in the second part of the sample. Our empirical analysis below aims to more formally characterize this negative correlation between economic uncertainty and presidential approval.

Table 1 reports descriptive statistics for the variables in the model. The average presidential approval rating is 51.2% in Brazil, 46.7% in Chile, 54.6% in Colombia, and 56.3% in Mexico. The estimates are more volatile in Brazil and Colombia, where the variable has standard deviations of 15.4% and 15.3%, respectively. In Chile and Mexico, these estimates are respectively 10.3% and 7%, indicating greater stability over time. The mean value of our proxy for economic uncertainty, the EPU index, is 119.3 in Brazil, 99.4 in Chile, 97.2 in Colombia, and 101 in Mexico. Thus, in our sample, Brazil exhibits the highest level of economic uncertainty, followed by Mexico, Chile, and finally by Colombia. It is worth noting that Brazil and Mexico have experienced episodes of high economic uncertainty with EPU index values as high as 473.5 and 428.7, respectively. In Chile and Colombia, the EPU index has risen to much lower maxima of 282.8 and 236.3, respectively.

**Table 1:** Descriptive statistics.

	Pres. Approval	EPU Index	Ind. Prod. Index	Inflation (%)	Unemp. (%)	Stock Ret. (%)
	***Brazil***					
Mean	51.2	119.3	95.8	7.5	9.3	1.4
Median	50.8	104.7	96.8	6.4	9.6	1.6
St. Dev.	15.4	70.6	12.8	4.7	2.7	8.5
Min.	21.8	14.6	72.9	1.6	4.6	-39.6
Max.	92.6	473.5	115.6	33.0	14.3	24.0
	***Chile***					
Mean	46.7	99.4	95.0	4.6	7.9	0.8
Median	46.4	89.1	96.4	3.8	7.6	0.5
St. Dev.	10.3	43.7	5.0	3.3	1.5	4.5
Min.	25.2	31.6	81.0	-3.4	5.5	-9.6
Max.	75.3	282.8	102.4	13.7	11.0	16.1
	***Colombia***					
Mean	54.6	97.2	85.6	6.8	11.8	0.4
Median	62.0	92.3	88.6	5.8	11.5	0.2
St. Dev.	15.6	31.0	12.0	4.7	2.2	4.9
Min.	20.9	41.1	61.5	1.8	7.3	-19.0
Max.	81.5	236.3	105.6	20.8	17.9	11.0
	***Mexico***					
Mean	56.3	101.0	96.6	8.0	4.0	1.3
Median	57.0	86.5	96.1	4.5	3.9	1.3
St. Dev.	7.0	71.7	8.9	8.2	1.0	6.3
Min.	38.9	8.5	74.7	2.1	2.1	-29.5
Max.	72.9	428.7	110.9	51.7	6.2	19.3

This table provides descriptive statistics for the variables included in the panel vector autoregressive (PVAR) analysis (presidential approval rating, the EPU index, industrial production index, inflation, unemployment rate, and stock index returns) in Brazil, Chile, Colombia, and Mexico.

Table 2 reports correlations between the EPU index and presidential approval ratings within and across countries. Panel A shows the contemporaneous relationship between the EPU index and presidential approval for each country in the sample. This correlation is -0.15 in Brazil, -0.40 in Chile, -0.36 in Colombia and 0.21 in Mexico. In three out of the four countries, the estimated correlations point towards a negative relationship between economic policy uncertainty and presidential approval. Panel B reports the correlations between the EPU index values for the four countries. The coefficients are significantly different from zero, which might be indicative of a common source of economic uncertainty in the region or even spillovers across countries (see, e.g., [42, 43]). Panel C also reports significant correlations between presidential approval ratings across the countries in the sample. For example, the correlation between ratings in Brazil and Colombia is 0.67, while in Chile and Mexico it is 0.39.

**Table 2:** Correlations.

	Panel A: Corr(EPU Index; Pres. Approval)			
Brazil		-0.154		
Chile		-0.399		
Colombia		-0.364		
Mexico		0.210		
	Panel B: EPU Index			
	Brazil	Chile	Colombia	Mexico
Brazil	1.00			
Chile	0.30	1.00		
Colombia	0.39	0.52	1.00	
Mexico	-0.20	0.32	0.36	1.00
	Panel C: Presidential Approval			
Country	Brazil	Chile	Colombia	Mexico
Brazil	1.00			
Chile	0.26	1.00		
Colombia	0.67	0.38	1.00	
Mexico	0.22	0.39	0.26	1.00

This table reports contemporaneous correlations for selected variables in the sample. Panel A shows the correlation between the EPU index and presidential approval ratings by country, whereas panel B (C) shows the correlations between the EPU indices (presidential approval ratings) across countries.

## 4. Empirical analysis

In this section, we empirically test whether economic uncertainty has an effect on presidential approval. We report results from two complementary empirical analyses. First, we re-estimate the model developed by [29] to study the relationship between presidential approval, economic variables, and countries’ political regimes and we include the EPU index in the analysis. Secondly, we estimate a macro-political dynamics model using a Panel-VAR approach to quantify the extent to which a shock in economic uncertainty impacts presidential approval.

### 4.1 An extended version of the model by [29])

In a recent paper, [29] study how policy regimes affect economic accountability in Latin America. The author’s empirical strategy involves examining whether the effect of economic variables on presidential approval varies across policy regimes. An additional variable allows them to explore the possibility of an interaction between economic growth and policy regime (either neoliberalism or statism). The rationale behind this exercise is that the relationship between presidential approval and economic growth captures the extent of economic accountability in a country. Therefore, if policy regimes mediate this relationship, then, it follows that they affect economic accountability. Using quarterly data from 18 Latin American countries, the authors conclude that in a neoliberal (statist) political regime, economic accountability is proportionally lower (higher) than that observed in countries with fewer neoliberal (statist) politicies.

Even though our focus is not on the effect of policy regimes on economic accountability, the authors’ framework allows us to straightforwardly incorporate our proxy for economic uncertainty into the model. Thus, we investigate the relationship of interest to us in this study and use all the control variables in the model by [29]. Given that the original study uses quarterly data, we take the quarterly average of the monthly EPU index. The EPU index is only available for four (Brazil, Chile, Colombia, and Mexico) of the 18 Latin American countries in [29]. Consequently, the number of observations in our empirical analysis is smaller than in [29]: 248 vs. 959 observations, respectively.

Table 3 shows our results. We estimate the same specifications as [29], but we add the EPU index (contemporaneous and lagged) as an additional regressor. In Model 1, on the one hand, the estimated coefficient for the EPU index is negative (-2.7), but it is not statistically significant. On the other hand, the estimated coefficient for the lagged EPU index is positive and statistically significant at the 90 percent confidence level. In models 2 to 6, policy regime variables are successively included and interacted with economic growth. In model 2, the estimated coefficient for the contemporaneous value of the EPU index is once again negative (-2.85), but it is now statistically significant at the 90 percent confidence level. The point estimate indicates that a higher level of economic uncertainty has a detrimental effect on presidential approval. The economic magnitude of these estimates is substantial as well. For example, a 70-point increase in the EPU index, which corresponds to a one-standard-deviation rise in Brazil and Mexico, reduces presidential approval by around 12 percentage points (-2.85*ln(70)). Considering that the mean presidential approval rating in the sample is 50 percent, overall, the effect of economic uncertainty is approximately 24 percent (0.24 = 12/50). The estimated coefficient for the lagged EPU index remains positive (2.25) as in model 1, but it is not statistically significant at standard confidence levels. We observe a similar pattern in the remaining models (3 to 6), with a negative and statistically significant estimate for the contemporaneous EPU index and a positive but statistically non-significant estimate for its lagged value.

**Table 3:** Economic uncertainty and presidential approval in the [29]’s model.

	Model 1	Model 2	Model 3	Model 4	Model 5	Model 6
**EPU t**	**-2.703**	-2.858*	-2.786*	-3.049*	-3.050*	-3.130*
	**(1.705)**	**(1.694)**	**(1.664)**	**(1.627)**	**(1.646)**	**(1.628)**
**EPU t-1**	2.909*	**2.259**	**1.884**	**2.568**	**2.426**	**2.402**
	**(1.737)**	**(1.719)**	**(1.701)**	**(1.692)**	**(1.689)**	**(1.688)**
Approval t-1	0.758***	0.744***	0.726***	0.738***	0.753***	0.741***
	(0.047)	(0.047)	(0.047)	(0.047)	(0.046)	(0.046)
Honeymoon t	11.76***	11.59***	11.52***	11.72***	11.377***	11.421***
	(2.326)	(2.316)	(2.281)	(2.288)	(2.309)	(2.277)
Scandal t	-0.98	-1.19	-0.94	-1.241	-1.048	-0.849
	(2.049)	(2.014)	(1.994)	(1.997)	(2.011)	(1.996)
Growth t	0.596***	0.530***	0.831***	0.440**	0.556***	0.703***
	(0.188)	(0.196)	(0.229)	(0.182)	(0.186)	(0.199)
Inflation t	-1.059*	-0.471	-0.58	-0.569	-0.168	-0.202
	(0.516)	(0.807)	(0.786)	(0.783)	(0.721)	(0.704)
Neoliberalism t		2.136+	2.987**	1.135		
		(1.2)	(1.188)	(1.256)		
Statism t		-8.588	-14.278	-5.267		
		(9.109)	(8.782)	(9.312)		
Growth x Neoliberalism t			-0.283***			
			(0.099)			
Growth x Statism t				0.363**		
				(0.166)		
Policy Regime t					1.502	1.392
					(0.934)	(0.88)
Growth x Policy Regime t						-0.187***
						(0.069)
Constant	16.43**	17.37**	18.27**	19.63***	18.45***	19.61***
	(7.987)	(7.793)	(7.826)	(7.601)	(7.818)	(7.63)
N	248	248	248	248	248	248
r2	0.74	0.75	0.74	0.76	0.75	0.75

This table shows multivariate regression estimates for the models reported by [29] and extended through inclusion of the EPU index. The sample includes Brazil, Chile, Colombia, and Mexico. See [29] for a detailed description of models and variables.

* p<0.10

** p<0.05

***p<0.01

Similarly to [29], we find that the lagged presidential approval, Honeymoon, Scandal, economic growth, and inflation variables have the expected signs and are statistically significant at the standard levels of confidence. We observe similar results, in terms of estimated signs and statistical significance, for the policy regime variable and its interaction with economic growth.

Overall, the results presented so far suggest that economic uncertainty has an adverse effect on presidential approval. This effect remains statistically significant upon including several control variables, including proxies of the stance of the economy. This indicates that the effect of economic uncertainty is different than the one produced by changes in macroeconomic aggregates.

### 4.2 A macro-political dynamics model with economic uncertainty

In our second empirical analysis, we estimate a macro-political dynamics model (see, e.g., [35, 44]), which includes an economic uncertainty variable in addition to the standard macroeconomic political variables (presidential approval). More specifically, we estimate a panel vector autoregressive (PVAR) model able to capture the joint dynamics of the variables in the system using information for several countries simultaneously (see [45]). Crucially, the method allows for the computation of impulse response functions (IRF), which indicate the extent to which a shock on one variable (i.e., economic uncertainty) impacts all other variables (in particular, presidential approval) in the system. It is worth mentioning that this method treats all the variables as endogenous. In the following subsection, we briefly describe the PVAR method before presenting our empirical results.

#### 4.2.1 The PVAR method

Following [45], the PVAR model is defined as follows:

$$y_{i,t}=B_{o}+B_{1}y_{i,t-1}+\mu_{i}+\varepsilon_{i,t},\;t=1,\ldots ,T;i=1,\ldots ,N.$$ (6)

where the vector *y*_*i*,*t*_ contains realizations at time *t* of the set of endogenous variables considered in the analysis for country *i*. In our models, we include the (log of the) EPU index, the unemployment rate, the industrial production index, and the inflation rate in this vector. In some specifications, we also include the stock index return. The vector ***μ***_*i*_ contains country fixed effects and the vector *ε*_*i*,*t*_ contains idiosyncratic errors. The selection of variables for vector *y*_*i*,*t*_ is based on prior literature (see, e.g., [35]).

The interpretation of the IRF estimates depends foremost on the order in which the variables are included in the vector *y*_*i*,*t*_, since it defines the degree of interdependence between the reduced-form PVAR residuals. Following prior literature (see, e.g., [27, 35]), we arrange the variables from most exogenous to most endogenous, as follows: the EPU index (our measure of economic uncertainty), the inflation rate, unemployment rate, and finally, the industrial production index. The central assumption behind this ordering is that uncertainty shocks initially and swiftly affect financial markets proxied for by the stock market index return. Then, the consequences of these shocks spill over into the real economy and affect inflation and unemployment rates, as well as, finally, the industrial production index.

It is important to note that this order, which has been widely used in prior literature, may be more reliable when either a macroeconomic or a financial-market proxy of uncertainty is used. Given that the EPU index tracks uncertainty-related economic policies that may be viewed as endogenous government responses to the state of the economy, the assumed order may be incorrect. As a robustness test, we estimate a version of the PVAR model in which the EPU index is the most endogenous variable. Following [27], we achieve identification of the IRF using a Cholesky decomposition of the variance-covariance matrix of the vector *y*_*i*,*t*_.

The PVAR model is estimated using the GMM method, as described in [46] and [45]. The method deals with the inclusion of both the lag of the dependent variable and country fixed effects by applying the forward orthogonal deviation transformation of [47]. This transformation subtracts the average of all available future realizations of a variable from all country-month observations to remove the fixed effects. Since this transformation does not use past variable realizations, they remain valid instruments in the GMM estimation.

We select the optimal lags for the PVAR model and the optimal number of lags used as instruments in the GMM estimation method (see details below) using the Hannan-Quinn information criteria (HQIC) proposed by [48]. The authors offer consistent model selection criteria for GMM models using [49]’s (1982) J statistic for over-identification restrictions. This statistic is particularly useful when dealing with an unbalanced panel such as the one used in this study. Based on this test, we select a PVAR model with one lag (k = 1) in the autoregressive part and three lags (*q* = 3) to build GMM moment conditions.

As mentioned above, the estimation of a PVAR model is suitable for several reasons. First, it naturally captures dynamic interlinkages between the variables in the system. Secondly, it treats all the variables as endogenous, which avoids having to comply with stringent exogeneity assumptions (which was the case in our first empirical analysis). Thirdly, it enables unobserved time-invariant heterogeneities across countries to be controlled for through the inclusion of country fixed effects.

#### 4.2.2 Results

**Fig 2:**
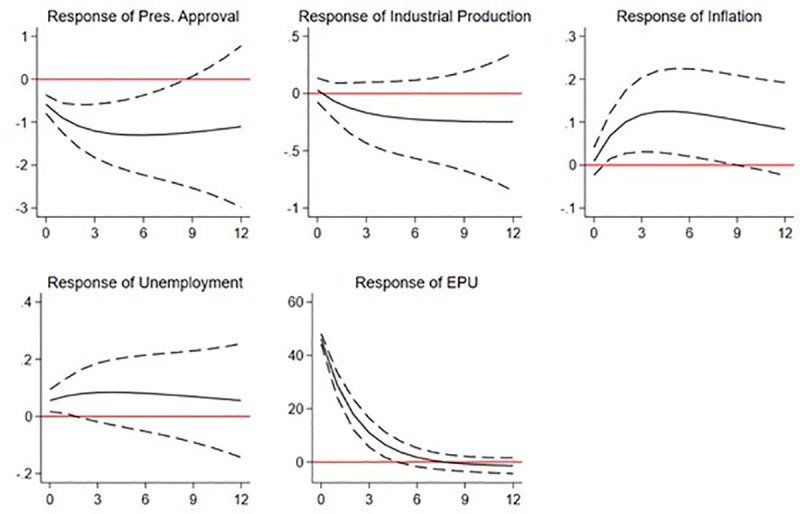
IRF of a shock on the EPU index. This figure shows plots reporting the responses (orthogonalized impulse response functions) of presidential approval ratings and a set of economic variables (industrial production, inflation, and unemployment rate) to a one-standard-deviation shock on the EPU index. IRF are estimated using a PVAR model with data from Brazil, Chile, Colombia, and Mexico. The EPU index is considered the most exogenous variable in the model.

**Fig 3:**
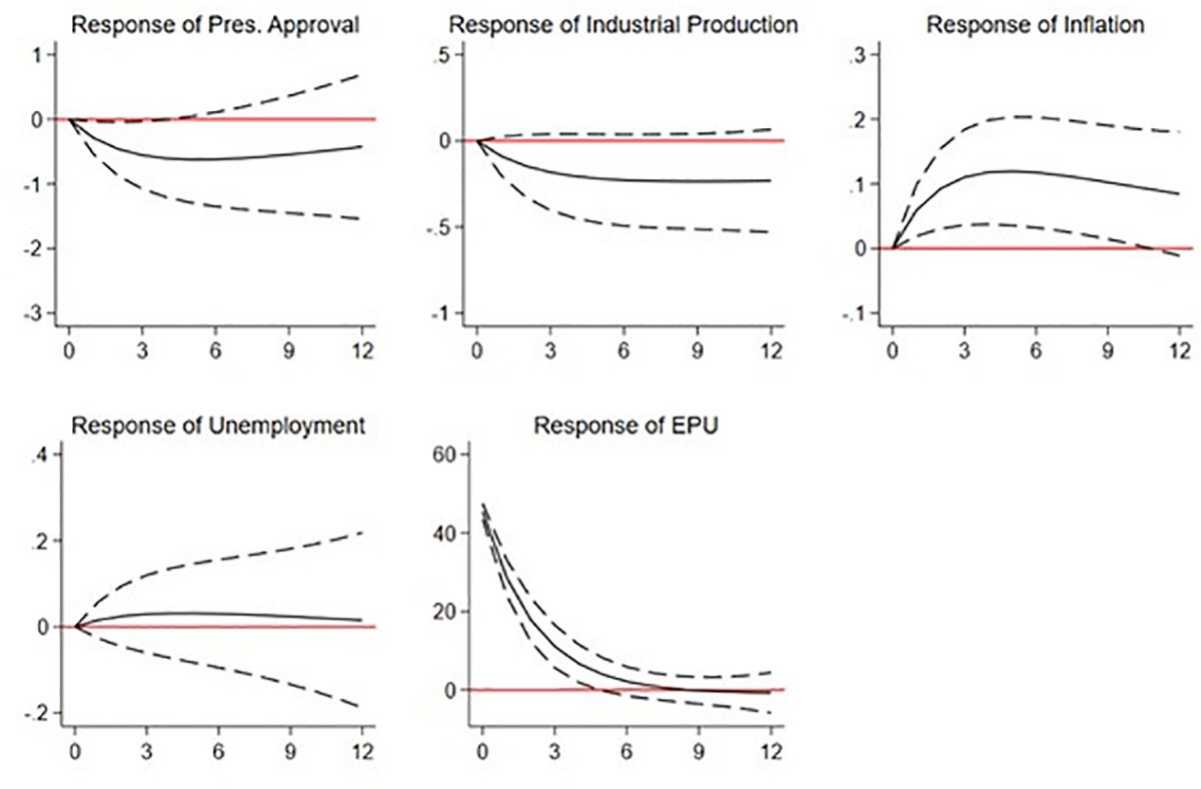
IRF of a shock on the EPU index. This figure shows plots reporting the responses (orthogonalized impulse response functions) of presidential approval ratings and a set of economic variables (industrial production, inflation, and unemployment) to a one-standard-deviation shock on the EPU index. IRF are estimated using a PVAR model with data from Brazil, Chile, Colombia, and Mexico. The EPU index is considered the most endogenous variable in the model.

Figs 2 and 3 show the estimated IRFs. In both figures, we include the EPU index, the inflation and unemployment rates, and the industrial production index in vector *y*_*i*,*t*_. However, whereas the EPU index ranks as the most exogenous variable in Fig 2, in Fig 3, we consider it to be the most endogenous. Both plots show the variable responses to a one-standard-deviation shock on the EPU index. Note that an uncertainty shock implies an increase in the level of the EPU index, i.e., greater economic uncertainty. We focus on the expected response for the presidential approval variable (see the upper-left plot in both figures).

In Fig 2, the estimated IRF shows a decrease in the level of presidential approval after an economic uncertainty shock, which is consistent with our results in section 4.2.1. The confidence interval indicates that the estimated effect on presidential approval is statistically significant and that it lasts for approximately nine months. We observe that the impact on presidential approval is strongest in month five, for which the point estimate is around -1.5. This evidence supports our previous results and points to a detrimental effect of economic uncertainty on presidential approval. When we look at the impact of the uncertainty shock on the other variables in the system, we find that it reduces industrial production (but not significantly) and significantly increases the inflation rate over the following six months. Finally, we observe a short-lived increase in the unemployment rate. All in all, the IRF function estimates show that economic uncertainty shocks significantly affect political variables and the overall economic outlook: inflation especially, and unemployment marginally. These results regarding the impact of uncertainty on economic variables are consistent with the theoretical framework described above and prior literature (see, e.g., [27, 50, 51]).

In Fig 3, we repeat the analysis but we position the EPU index as the most endogenous variable. We still find that a shock in economic uncertainty greatly reduces presidential approval and this effect remains statistically significant. Compared to the results in Fig 2, we now find that the estimated effect on presidential approval is shorter (5 months vs. 9 months). As regards the other variables, we observe that industrial production also declines, although this effect is not statistically significant. As before, we estimate a positive impact on the inflation rate and a mild effect on the unemployment rate as compared with the estimates reported in Fig 2. With this specification, we find that the unemployment rate increases in the three months following the shock, but the effect is not statistically significant. Overall, the results in Fig 3 also suggest that an economic uncertainty shock has a detrimental effect on presidential approval, even when the model is re-specified with the EPU index as the most endogenous variable. Additionally, we document effects on the real economy primarily through a temporary increase in the inflation rate.

It is worth highlighting the strong estimated effect of the EPU index on inflation in both figures. This effect is fully consistent with the model of [2] which is used as a theoretical framework in this study and according to which an EPU shock is strongly associated with higher unexpected inflation. The authors’ model predicts that, once the voters observe this inflation surprise, the incumbent’s re-election chances decrease.

Finally, in unreported results available upon request from the authors, we find that our estimations are robust to the inclusion in the model of the stock index returns variable. Prior literature (see, e.g., [32, 33]) has used this variable as a proxy for market uncertainty, so including it in the model reduces potential bias in our baseline estimates.

## 5. Conclusions

In this study, we examine the extent to which economic policy uncertainty affects presidential approval ratings. Political economy models (see, e.g., [1, 2]) have established that a country’s economic outlook is intrinsically linked to its politics. A large body of literature testing the impact of economic variables on presidential approval has yielded mixed results (see, e.g., the surveys by [3, 4]), but the corresponding effects of EPU have not been examined. We investigate this relationship empirically in four Latin American countries (Brazil, Chile, Colombia, and Mexico) for which comparable data is available, using the newspaper-based economic policy uncertainty index of [27] and presidential approval ratings from the Executive Approval Database (EAD) 1.0 (see [28]).

We perform two complementary empirical analyses. First, we extend the empirical model built by [29] to study the relationship between presidential approval, economic variables, and political regimes. Upon adding the EPU index to their model, we find that the estimated coefficient for this variable is negative and statistically significant, indicating that higher uncertainty reduces presidential approval. In the second analysis, we estimate a set of impulse response functions derived from a panel vector autoregressive (PVAR) model including economic variables and presidential approval. We find that an uncertainty shock significantly reduces presidential approval as well. A one-standard-deviation increase in the uncertainty index reduces presidential approval by approximately 12 percent.

Our results are consistent with the theoretical political economy model of [2]. In this model, uncertainty in economic policy is captured by the policymaker setting an inflation rate that is higher than the one expected by the voters. Upon registering this higher rate, the voters update their confidence in the future competence of the incumbent (the policymaker), thereby reducing the latter’s re-election chances. More broadly, if we take into account prior evidence (see, e.g., [27]) documenting a negative correlation between the EPU index and output growth, the model of [2] also predicts a downgrade in the voters’ forecast of the incumbent’s future competence. An additional channel through which EPU may impact presidential approval would involve the competence signal of the [2] model being affected. However, it is unclear why this would be the case as it is neither possible to determine whether the EPU shock is domestic or foreign, nor possible to ascertain whether it could impact the country’s policy regime. These two mechanisms are discussed in the literature as ways of distinguishing between competence shocks and non-political shocks (see, e.g., [30, 31, 52]). This is an issue for future research.

We contribute to the literature by providing new empirical evidence on an understudied topic: the effect of economic uncertainty on presidential approval ratings. We also contribute to the literature by focusing our study on a set of Latin American countries in which economic uncertainty is higher than in developed economies [6]. Finally, we contribute to the literature on macro-political dynamics by studying the interlinkages between economic and political variables using a PVAR model that captures the dynamics among variables and incorporates several countries in the analysis simultaneously.

Overall, we have shown that both a country’s economic outlook and economic policy uncertainty play a significant role in presidential approval ratings in Latin America.

## Supporting information

S1 File (ZIP)
