# Supplementary material for: Economic policy uncertainty and presidential approval: Evidence from Latin America
Source: PLoS One. 2021 Mar 15;16(3):e0248432. doi: 10.1371/journal.pone.0248432 (PMC7959373; doi:10.1371/journal.pone.0248432)
Supplement: S1 File — (ZIP) [file pone.0248432.s001.zip › datacode/readme.docx]

**Data and Code for**

**“Economic Policy Uncertainty and Presidential Approval: Evidence from Latin America”**

**by Myriam Gómez-Méndez and Erwin Hansen**

**February 2021**

The zip file contains the following four files to replicate figures and regression results:

**Figures (correlations and impulse response functions, IRF)**

- Figures.dta: Stata database
- Figures_code.do: Stata do file

**Table 3 (main regression results)**

- Table3.dta: Stata database
- Table3_code: Stata do file
